# Influence of Machining, Polishing, and Glazing on Surface Properties and Biological Performance of Zirconia and Lithium Disilicate Dental Ceramics

**DOI:** 10.3390/jfb16110400

**Published:** 2025-10-27

**Authors:** Youngchae Cho, Min-Gu Cho, Jeong-Hyun Ryu, Ji-Yeong Kim, Sung-Hwan Choi, Hyungjoon Shim, Min-Ho Hong, Deuk Yong Lee

**Affiliations:** 1 Department of R&D Center, Hass Co., Ltd., 60, Haan-ro, Gwangmyeong 14322, Republic of Korea; yccho@hassbio.com (Y.C.); mgcho@hassbio.com (M.-G.C.); 2 Department of Orthodontics, Institute of Craniofacial Deformity, Yonsei University College of Dentistry, 50-1 Yonsei-ro, Seodaemun-gu, Seoul 03722, Republic of Korea; jhryu@yuhs.ac (J.-H.R.); katekim826@yuhs.ac (J.-Y.K.); selfexam@yuhs.ac (S.-H.C.); 3 BK21 FOUR Project, Yonsei University College of Dentistry, 50-1 Yonsei-ro, Seodaemun-gu, Seoul 03722, Republic of Korea; 4 Center for Systems Biology, Massachusetts General Hospital, 185 Cambridge Street, Boston, MA 02114, USA; 5 Department of Dental Biomaterials and Research Institute of Oral Science, College of Dentistry, Gangneung-Wonju National University, Gangneung 25457, Republic of Korea; tony6533@gmail.com (H.S.); mhong@gwnu.ac.kr (M.-H.H.)

**Keywords:** lithium disilicate (Li_2_Si_2_O_5_), zirconia, polishing, glaze, wettability, cytocompatibility, biofilm

## Abstract

Surface treatments play a crucial role in modifying the surface properties and biological performance of dental ceramics. This study investigated the effects of surface conditions on the wettability, cytocompatibility, and bacterial resistance of 4 mol% Y_2_O_3_-stabilized tetragonal zirconia polycrystal (4Y–TZP) and two lithium disilicate (Li_2_Si_2_O_5_) glass ceramics (Amber^®^ Mill (AM) and Amber^®^ Mill Abut-Crown (AC)). Human gingival fibroblast (HGF-1) responses and biofilm formation on the machined, polished, and glazed samples were evaluated. The polished 4Y–TZP sample exhibited the highest water contact angle (WCA; 71.3°), while that of the AC samples decreased as the sample was machined (58.4°), polished (46.8°), and glazed (14.0°). The wettability, cytocompatibility, and bacterial resistance of the dental ceramics were significantly influenced by material type and surface condition. Among the surface-treated samples, the glazed specimens exhibited the lowest WCA and bulk density; thus, wettability is an important factor for cell proliferation and bacterial resistance. Among all samples, HGF-1 cells adhered well to the glazed ceramics and significantly proliferated over time. Particularly, the 4Y–TZP and AC glazed samples exhibited the lowest biomass and strong resistance to biofilm formation and bacterial adhesion. Thus, the glaze dramatically affected HGF-1 cell growth and antibiofilm formation.

## 1. Introduction

Owing to their appealing aesthetics and excellent biocompatibility, metallic materials have been increasingly replaced by all-ceramic prosthetics as the preferred dental material for a wide range of applications, from veneers for single-tooth restorations to crowns and bridges [1,2,3,4,5,6,7,8,9]. Yttria-stabilized tetragonal zirconia polycrystal (Y–TZP) exhibits excellent mechanical properties, such as strength (>450 MPa) and fracture toughness (4–9 MPa·m^1/2^), rendering it widely used in hip joints and dental abutments [8,9,10,11,12,13]. Notably, as the yttria and/or additive (e.g., Al and La) content of zirconia increases, the aesthetic translucency dramatically increases [8,9]. Glass ceramics are more aesthetically appealing than ZrO_2_, which is susceptible to low-temperature degradation, due to the latter’s whiteness and opacity [1,3,8,9]. In addition to their biocompatibility and wear-resistant properties, glass ceramics offer high compressive strength and thermal expansion properties similar to those of natural teeth [1,2,3,4,5]. In particular, lithium disilicate (LD; Li_2_Si_2_O_5_) ceramics are widely preferred owing to their excellent fluorescence, opalescence, processability, and mechanical properties [5]. They have less edge chipping than ZrO_2_ and an appearance similar to that of natural teeth [1,2,3,4,5,8]. LD glass ceramics are favored in dentistry because they require less milling time and offer better aesthetics than zirconia crowns. In clinical practice, zirconia and LD have been used as implant abutment and crown materials, respectively [1,2,3,4,5,6,7,8,9].

The most important aspect of dental prosthetics is to enhance cell adhesion to the surface, which is in contact with gum tissue. However, they must also prevent bacterial cell adhesion to natural teeth or implants. Aesthetic dental ceramics are generally known for their low adhesion, and the lower the bacterial adhesion of biomaterials, the greater their ability to inhibit biofilm formation on the tooth surface in the oral cavity [10,11,12,13,14,15,16]. Surface treatment influences cellular responses to oral implant biomaterials [10,11,12,13], and surface roughness and wettability are key factors governing cell adhesion and proliferation [5]. Compared to machined ceramics, polished ceramics display reduced roughness and increased hydrophobicity, promoting protein adsorption and cell attachment [14,15,16]. Conversely, hydrophilicity significantly increases due to roughness-independent glaze treatment, thereby reducing protein adsorption [14,16]. Thus, glazed dental crowns are widely used in clinical practice and are commercially available because the machined surface is not exposed to the oral cavity without polishing or glazing.

From the perspective of a clinician, using an oral scanner enables the fabrication of a ceramic crown with excellent marginal and internal fit [17]. Prostheses fabricated using dental CAD/CAM systems have clinically acceptable marginal fit values of <100 μm. Lee et al. [18] reported that the marginal fit values of three types of commercial ceramic blocks (Hass Rosetta, IPS e.max CAD, and VITA Suprinity) are within clinical limits (<100 μm). Blocks with marginal fit values of >120 μm are clinically unacceptable for prostheses owing to periodontitis, cement dissolution, and secondary caries [17,18]. LD ceramic crowns prepared using an intraoral scanner or existing methods are satisfactory in both marginal and internal fits, with higher clinician satisfaction [17]. In this study, the marginal fit was not considered.

**Figure 1:**
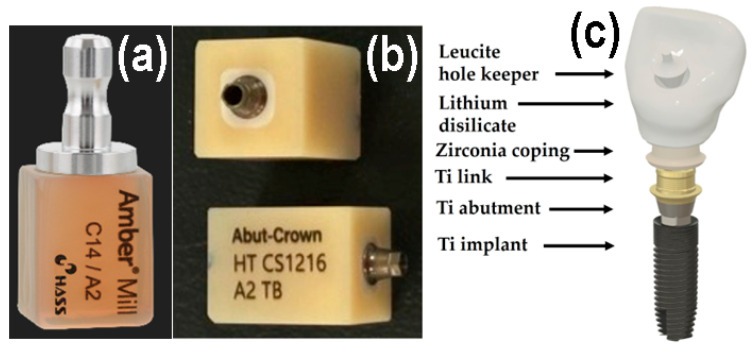
Photographs of (**a**) AM, (**b**) AC. (**c**) Schematic of a dental crown prepared using AC.

The all-in-one dental ceramic block Amber^®^ Mill Abut–Crown (AC; CS1216 A2 TB, Hass Co., Ltd., Gangneung, Gangwon–do, Republic of Korea) comprises a Ti abutment and link, ZrO_2_ ceramic coping, pre-crystallized machinable LD glass ceramic, and a leucite ceramic for the hole keeper, as depicted in Figure 1. Metallic-colored Ti abutments cause soft tissue contraction and gingival recession and are visible through thin bone and mucosa. Therefore, ivory-colored ZrO_2_ copings, which closely match the color of natural teeth, are often used [1,12,19]. The upper surface of the LD crown should be easy to clean and should not require gingival cell attachment or proliferation for an aesthetic crown [1]. Conversely, the long-term retention of dental glass ceramic crowns on the buccal side depends on strong gingival cell adhesion beneath the epithelial–junctional junction [5,17]. Polishing reduces surface roughness, while glazing creates better conditions for cell viability and proliferation. Moreover, commercial dental ceramics have excellent biocompatibility. Despite these considerations, few studies have explored the adhesion between different surfaces of ceramics and human gingival fibroblasts (HGF-1s) [5,20].

To characterize the biological performance of dental ceramics, both cell proliferation and bacterial surface attachment should be simultaneously assessed [20]. Bacterial adhesion refers to the adsorption of salivary-derived proteins, which can mediate bacterial attachment and biofilm formation [20,21,22,23]. In this study, the bulk density, apparent porosity, water contact angle (WCA), roughness, cytotoxicity, and cell proliferation of ZrO_2_ and LD glass ceramics were investigated according to surface treatments, which included machining, polishing, and glazing, to elucidate cellular responses to dental ceramics. Bacteria involved in the initial colonization of the tooth surface contribute to biofilm formation. Thus, the salivary biothickness and biomass of these biofilms were evaluated using human saliva. The null hypothesis is that material type and surface conditions do not affect the bulk density, porosity, WCA, roughness, cytocompatibility, and bacterial resistance of ZrO_2_ and LD glass ceramics.

## 2. Materials and Methods

### 2.1. Sample Preparation

**Figure 2:**
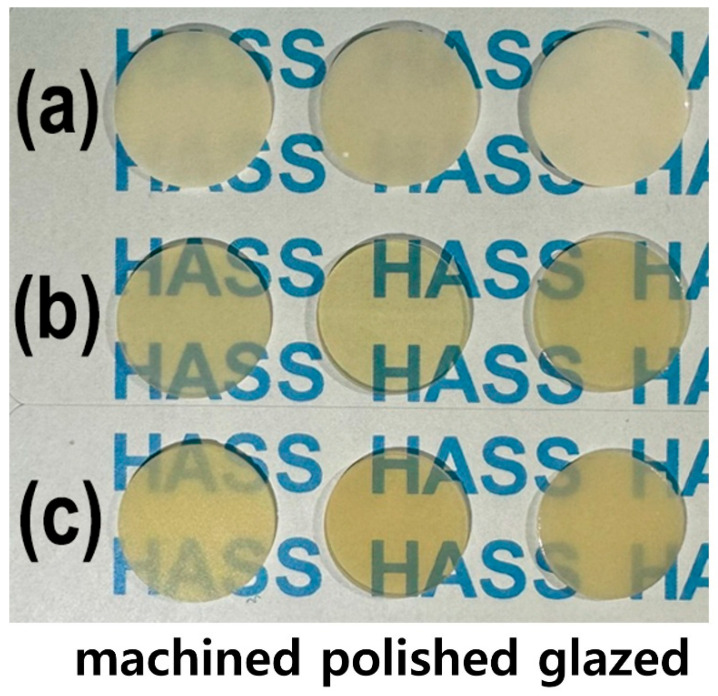
Photographs of the machined, polished, and glazed samples: (**a**) 4Y–TZP, (**b**) AM, and (**c**) AC.

As shown in Figure 1, the LD glass ceramic Amber^®^ Mill (AM) blocks (Hass Co., Ltd., Gangneung, Republic of Korea) and pre-crystallized AC blocks comprised SiO_2_ (62–75%), Li_2_O (20–30%), Al_2_O_3_ (<5%), P_2_O_5_ (1–10%), and other oxides and pigments (0–10%) [2]. After pre-sintering at 750 °C for 40 min, AM was processed and then subjected to secondary sintering at 815–855 °C for 15 min (Figure 1a). AC was subjected to primary crystallization using a single-stage sintering at 840 °C for 15 min. The crystallinity of LD was approximately 60–70% [2], and no additional sintering was required for AC. Fully crystallized 4 mol% Y_2_O_3_–stabilized tetragonal ZrO_2_ polycrystal (4Y–TZP) blocks (Razor 1100) were provided by UNC International Co., Ltd. (Seoul, Republic of Korea). All samples were cut into disc shapes with a diameter and thickness of 12 and 0.6 mm, respectively. The as-prepared disc specimens (as-machined, untreated) were selected as controls. These discs were ground using diamond mesh #230 (HRG–150, AMTechnology, Asan, Chungbuk, Republic of Korea), polished with a 1 μm diamond paste (SPL–15 Grind–X, Okamoto Co., Annaka, Gunma, Japan), and glazed to their final crystallized form, as depicted in Figure 2. Sandblasting samples were prepared by blasting alumina beads (particle size 50 μm) using a dental sandblaster (Basic eco, Renfert GmbH, Hilzingen, Germany) at a pressure of 2–2.5 bar and an angle of 70 degrees, maintaining a distance of approximately 10 mm for 20 s. The polished samples were sandblasted and sonicated, then glazed at 760–800 °C for 1 min using an InSync^®^ stain and glaze paste kit (Jensen Dental, North Haven, CT, USA) [24]. Subsequent cooling occurred at low temperatures to prevent the induction of failure from occurring on the surface.

### 2.2. Surface Characterization

#### 2.2.1. Bulk Density and Apparent Porosity

The bulk density (*ρ_b_*) of the glass ceramics (*n* = 5) was determined using the Archimedes equation [25], given in Equation (1), where *W*_1_, *W*_2_, *W*_3_, and *ρ_w_* are the weight of the dried sample, weight of the sample immersed in distilled water (DW) after it was boiled in DW for 3 h and cooling to 25 °C, water absorption weight in the air after wiping off the water on the sample surface, and density of DW, respectively. The apparent porosity (π, *n* = 5) was determined using Equation (2) [25].

(1) $$\rho_{b}=\frac{W_{1}}{W_{3}-W_{2}}\times \rho_{w}$$

(2) $$\pi =\frac{W_{3}-W_{1}}{W_{3}-W_{2}}\times 100$$

#### 2.2.2. WCA and Roughness

The WCA (*n* = 5) was measured using a droplet analyzer (SmarDrop Standard, FEMTOFAB, Seongnam, Gyeonggi-do, Republic of Korea). DW (2 μL) was added to each sample, and after 5 s, the static contact angle was measured and analyzed [26,27,28]. The arithmetic average roughness (*Ra*) of the samples (*n* = 5) was measured using a roughness meter (Surftest SJ-310, Mitutoyo, Kawasaki, Japan). To determine *Ra*, a stylus profilometer (cut–off length: 0.8 mm, evaluation length: 2.4 mm, traversing speed: 0.5 mm/s, returning speed: 1 mm/s, tip radius: 2 μm) was used to measure the disc specimens five times at 120° intervals. To demonstrate the three-dimensional nature of surface roughness (*n* = 5), the arithmetic mean surface height (*Sa*), average maximum height of the profile (*Rz*), and maximum height (*Sz*) of the samples with various surface modifications were determined using an atomic force microscope (AFM; Aqua-ILM, NanoMagnetics Instruments Ltd., Oxford, UK). AFM was performed in both the dynamic and contact modes, scanning an area of 10 µm × 10 µm at a speed of 5 µm/s in a point-by-point manner [29]. Prior to analysis, the AFM images were plane-leveled and detrended to remove background curvature.

### 2.3. Cytocompatibility

#### 2.3.1. Cytotoxicity and Cell Proliferation

Before incubation, the samples were cleaned in an ultrasonic bath containing acetone for 10 min, rinsed with DW for 10 min, and then autoclaved for 15 min at 121 °C. The cytotoxic potential (*n* = 5) was assessed by performing extract assays for LD and 4Y–TZP according to International Organization for Standardization (ISO) 10993–5 [28]. HGF-1 (NCTC Clone 929; ATCC, Manassas, VA, USA) and L–929 (Korea Cell Line Bank, Seoul, Republic of Korea) cells were employed for analyses. The extract was aseptically collected from the medium at a sample–to–extraction medium ratio of 0.2 g/mL (ISO 10993–12) [26,27,28]. The single-strength minimum essential medium (1X MEM, Dulbecco’s Modified Eagle’s Medium, Thermo Fisher Scientific, Gibco, Waltham, MA, USA) with 10% fetal bovine serum (Thermo Fisher Scientific, Gibco, Waltham, MA, USA) and 1% penicillin–streptomycin (Thermo Fisher Scientific, Gibco, Waltham, MA, USA) was used. A 96-well plate was incubated at 37 °C in a 5% CO_2_ atmosphere. The test extracts were applied to three separate confluent monolayers of L-929 and HGF-1 cells grown in a CO_2_ incubator for 24 h. The reagent, negative (high-density polyethylene film, RM-C, Hatano Research Institute, Hadano, Japan), and positive (polyurethane film, RM-A, Hatano Research Institute, Hadano, Japan) controls were each cultured in triplicate and applied to confluent L-929 and HGF-1 monolayers. All monolayers were incubated at 37 °C in the presence of 5% CO_2_ for 48 h. After incubation, the morphological changes of the cells were examined, and biological responses were evaluated using an inverted microscope (Eclipse Ts2, Nikon, Tokyo, Japan) and the iMark microplate absorbance spectrophotometer (Bio-Rad, Hercules, CA, USA) [26,27,28]. Water-soluble tetrazolium salts (WSTs) represent a series of different water-soluble dyes for MTT assays, which can provide different absorbance spectra of the formed formazans, and EZ-cytox (EZ-3000, Dogenbio Co., Ltd., Seoul, Korea) can yield directly readable water-soluble formazan. For quantification, the absorbance of the stained solution was measured at a wavelength of 415 nm using a microplate absorbance spectrophotometer. The value of the untreated cells (control sample, only cultured with culture medium) was set at 100%, and the cell viability of the treated cells was expressed as the percentage of the control sample [26,27,28].

Cell counting kit-8 (CCK-8; Dojindo Molecular Technologies, Inc., Tokyo, Japan) was used for the cell proliferation assay [26,27,28]. CCK-8 is nonradioactive, enabling a sensitive colorimetric assay for measuring the number of viable cells in cell proliferation. WSTs are reduced by intracellular dehydrogenases, yielding an orange product (formazan) that is soluble in tissue culture medium. The amount of formazan dye produced by intracellular dehydrogenases is directly proportional to the number of viable cells. The 96-well plate containing 100 μL of cell suspension (5 × 10^3^ cells/well) was incubated at 37 °C in a 5% CO_2_ atmosphere for 24 h. Test extracts (10 μL) were added to the plates, which were placed in an incubator for the appropriate periods (12, 24, 48, and 72 h). After adding 10 μL of CCK-8 solution to each well of the plate, the plate was incubated for an additional 2 h. For quantification, the absorbance of the coloring solution was measured at a wavelength of 450 nm using the microplate absorbance spectrophotometer [26,27,28]. All experiments were performed in five replicates, and the results are expressed as mean ± standard deviation.

#### 2.3.2. Live/Dead Cell Assay

**Figure 3:**
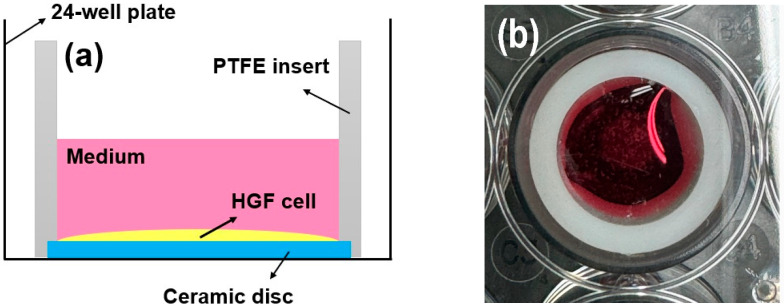
(**a**) Schematic and (**b**) photograph of the cell adhesion experiments using PTFE inserts mounted on rigid ceramic discs.

Following culturing for 72 h, the proliferative activity of the cells (*n* = 5) was investigated using a live/dead assay kit (Thermo Fisher Scientific, Waltham, MA, USA) [26,27,28]. Cell seeding (2 × 10^4^ cells/well) was performed using an efficient seal (cylindrical polytetrafluoroethylene (PTFE) insert: 2 mm thick, 15.5 mm high, 15 mm in diameter) around the ceramic disc. This approach is reliable for investigating the behavior of rigid biomaterials [26,27,28,30,31]. The HGF-1 cells were easily fixed onto a sample disc, and cell proliferation and spreading were evaluated [31]. Green indicated living cells, while red indicated dead cells [26,27,28]. As shown in Figure 3, a bevel with a height and thickness of 1 mm each was prepared from the bottom of the cylinder and fixed to the disc sample with a light frictional force. The disk insert system was applied to a 24-well plate [31]. After removing the PTFE insert from the disc, viable and dead HGF-1 cells were stained according to the manufacturer’s instructions and visualized using a fluorescence microscope (DP73; Olympus, Tokyo, Japan) [26,27,28,30,31].

### 2.4. Biofilm Analysis

#### 2.4.1. Preparation of Multispecies Human Salivary Biofilm

Referring to previous studies [20,21,22,23,32], human saliva was collected from healthy adult donors who had no active caries or periodontal disease and had not consumed antibiotics within the previous three months (IRB No. 2-2024-0005). To minimize oral hygiene and dietary influences, participants refrained from brushing their teeth for 24 h and abstained from food and drink for at least two hours prior to saliva collection. Human saliva was obtained from ten individuals and subsequently pooled in equal proportions to prepare a mixed saliva sample. This sample was then diluted to a concentration of 30% in sterile glycerol and stored at −80 °C for use as a biofilm model. The biofilm model was cultured in McBain broth medium (type II, porcine, gastric) (2.5 g/L), tryptone (2.0 g/L), bacteriological peptone (2.0 g/L), yeast extract (1.0 g/L), NaCl (0.35 g/L), CaCl_2_ (0.2 g/L), KCl (0.2 g/L), cysteine hydrochloride (0.1 g/L), haemin (0.001 g/L), and vitamin K1 (0.0002 g/L) [20,21,22,23,32]. The specimen was inoculated by mixing 1.5 mL of the McBain medium with 30 μL of mixed saliva, followed by culturing at 37 °C in a 5% CO_2_ environment. The nutrients were replaced by removing the existing medium every 24 h, and the biofilm was cultured in the same environment for a total of 72 h.

#### 2.4.2. Biofilm Thickness and Biomass Measurement

To remove loosely adhered microorganisms, the specimens (*n* = 5) were gently rinsed twice with phosphate-buffered saline (PBS). Subsequently, the viability of the attached biofilm was assessed via staining with a live/dead bacterial viability kit (Molecular Probes, Eugene, OR, USA) according to the manufacturer’s protocol [32]. Equal amounts of Syto 9 dye and propidium iodide, which stain live and dead bacteria, respectively, in the kit were mixed well. The mixture was then mixed with PBS in a ratio of 3 μL:1 mL, and 1 mL of each was dispensed into the samples. The biofilm was visualized at randomly selected locations using a confocal laser scanning microscope (LSM980, Carl Zeiss, Thornwood, NY, USA). Axial stacked biofilm images were captured, and the biofilm thickness was calculated using the system’s software (Zen, Carl Zeiss, Thornwood, NY, USA). The average biomass was determined using the COMSTAT plug-in (Technical University of Denmark, Kongens Lyngby, Denmark) and ImageJ (version 1.54, NIH, Bethesda, MA, USA) software [20,21,22,23,32].

### 2.5. Statistical Analysis

The values are expressed as mean ± standard deviation, and *p* < 0.05 was considered statistically significant. Two independent variables were considered: material type (4Y–TZP, AM, and AC) and surface treatment (machining, polishing, and glazing). Homogeneity of variance was assumed, and statistical analysis was performed using a two-way analysis of variance (ANOVA) followed by Tukey’s honest significant difference post hoc test regarding physical properties. In vitro and ex vivo assays were performed using a one-way ANOVA followed by Tukey’s post hoc analysis using the IBM SPSS software (version 23.0, IBM Co., Armonk, NY, USA) [20,21,22,26,27,28,32].

## 3. Results

### 3.1. Bulk Density and Apparent Porosity

**Figure 4:**
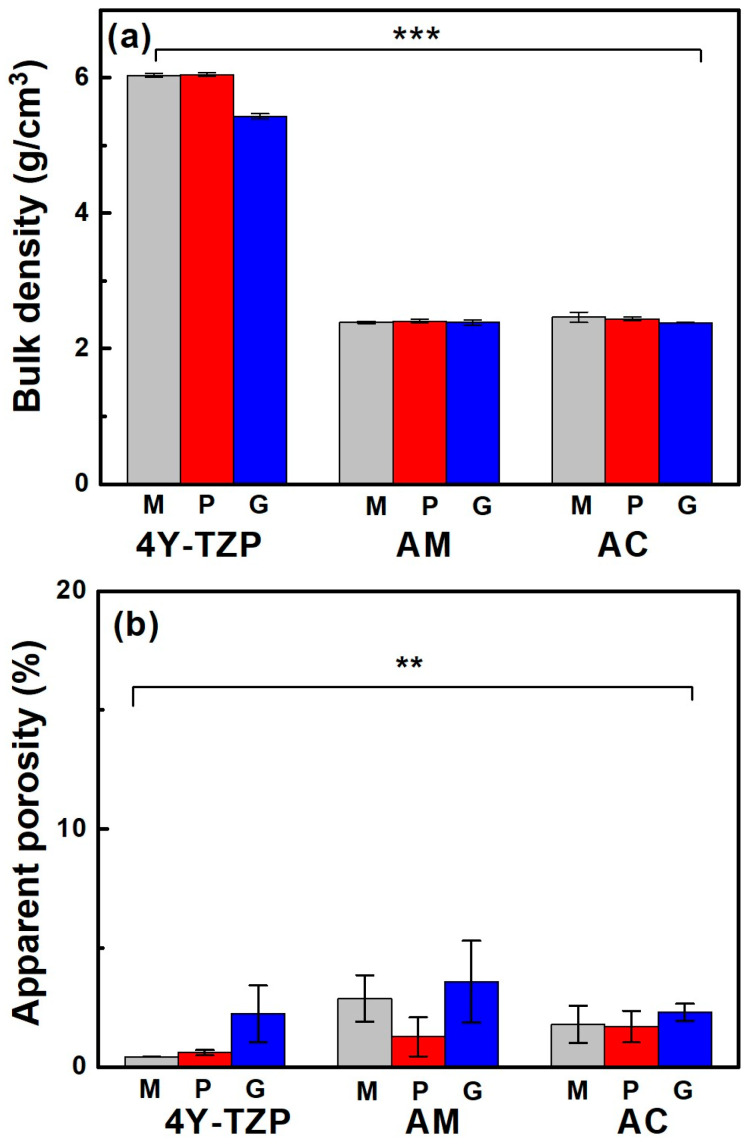
(**a**) Bulk density and (**b**) apparent porosity of various samples. M, P, and G represent machined, polished, and glazed, respectively. Statistical significance is indicated by ** (*p* < 0.01) and *** (*p* < 0.001).

When the samples were glazed, their bulk density (Figure 4a) and apparent porosity (Figure 4b) decreased and increased, respectively, regardless of the sample type. The bulk density of fully densified 4Y-TZP was dramatically reduced from 6.04 to 5.42 g/cm^3^ after glazing, which was attributed to the less rigid structure containing the glassy phase of the glaze [33], as summarized in Table 1. Bulk density was influenced by material type (partial eta-squared, $\eta_{p}^{2}=1.0$, *p* < 0.001) and surface condition ($\eta_{p}^{2}=0.928$, *p* < 0.001), as well as the interaction between material type and surface condition ($\eta_{p}^{2}=0.947$, *p* < 0.001). The Tukey’s post hoc analysis revealed significant differences between 4Y-TZP and LD ceramics (AM and AC) and between polishing (or machining) and glazing, with *p* < 0.001 for each. However, no dramatic differences in bulk density were determined between machining and polishing, AM and AC (*p* > 0.05). Thus, material type had a greater effect on bulk density than the surface condition.

Porosity was measured because the glaze had a lower specific gravity than 4Y-TZP and LD, which could reduce the density of the product. The increase in porosity qualitatively indicated that the microstructure of the glazed samples was more loosely bound than the other samples. Apparent porosity was influenced by material type ($\eta_{p}^{2}=0.409$, *p* < 0.01) and surface condition ($\eta_{p}^{2}=0.426$, *p* < 0.01) and was not affected by the interaction between material type and surface condition ($\eta_{p}^{2}=0.228$, *p* = 0.296). Thus, the null hypothesis that material type and surface treatment do not affect the bulk density and apparent porosity of ZrO_2_ and LD glass ceramics was rejected. Conversely, differences in the bulk density and apparent porosity of 4Y-TZP and LD glass ceramics (Figure 4b) were statistically significant. Moreover, surface condition (glaze) had a greater impact on apparent porosity than material type.

### 3.2. WCA and Roughness

**Figure 5:**
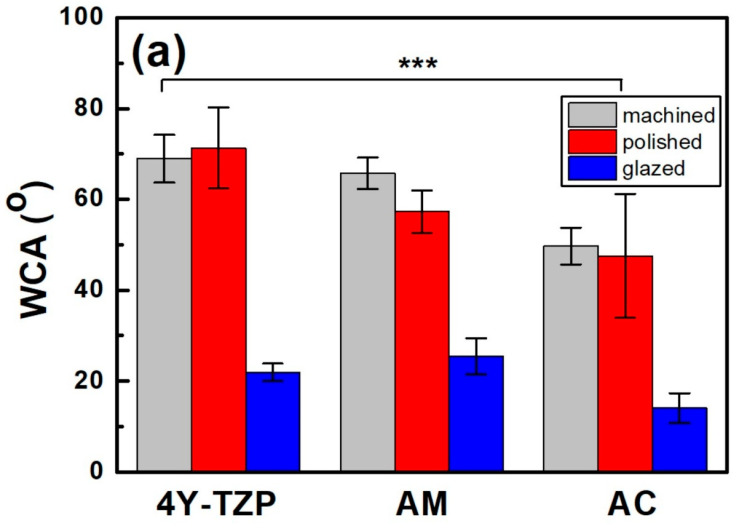

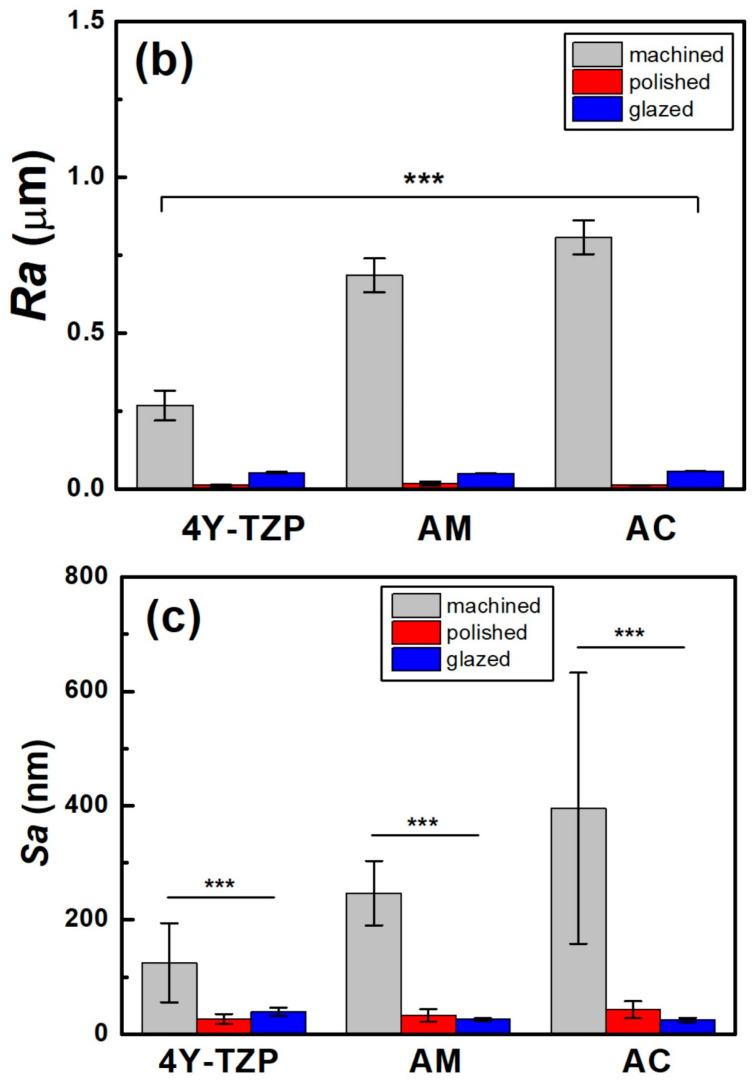
Changes in the (**a**) WCA, (**b**) *Ra*, and (**c**) *Sa* of various samples according to surface condition. Statistical significance is indicated by *** (*p* < 0.001).

After polishing, the WCA of 4Y–TZP increased from 68.9° to 71.3°, indicating a slight increase in hydrophobicity (Figure 5a). However, when 4Y–TZP was glazed, the WCA dramatically decreased to 21.9°, implying that the hydrophilicity of 4Y–TZP was significantly enhanced [15,16,24,34,35,36]. Thus, surface treatment had a significant effect on the WCA of ZrO_2_ and LD glass ceramics (*p* < 0.001). The WCA was influenced by material type ($\eta_{p}^{2}=0.711$, *p* < 0.001) and surface condition ($\eta_{p}^{2}=0.929$, *p* < 0.001; Figure 5a) but not by the interaction between material type and surface condition ($\eta_{p}^{2}=0.364$, *p =* 0.073). Polished 4Y–TZP had the highest WCA (71.3°) and lowest roughness. The difference in the WCA between machining and polishing was not significant (*p* = 0.536); however, polished ceramics (4Y–TZP, AM, and AC) exhibited different WCAs. Representing the hydrophilicity index, the WCA of 4Y–TZP decreased in the following order: polishing, machining, and glazing. However, LD exhibited a WCA decrease in the order of machining, polishing, and glazing [33]. The WCA of the AM samples gradually decreased from 62.8°, 56.6°, and 25.4° as the samples were machined, polished, and glazed, respectively. Moreover, the polished 4Y–TZP exhibited the highest WCA, whereas glazed AC had the lowest WCA. As shown in Figure 5, all samples showed similar results, with a dramatic decrease in WCA after glaze treatment.

**Figure 6:**
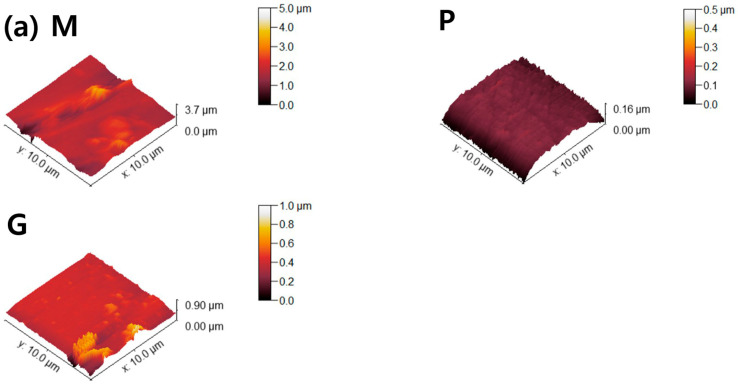

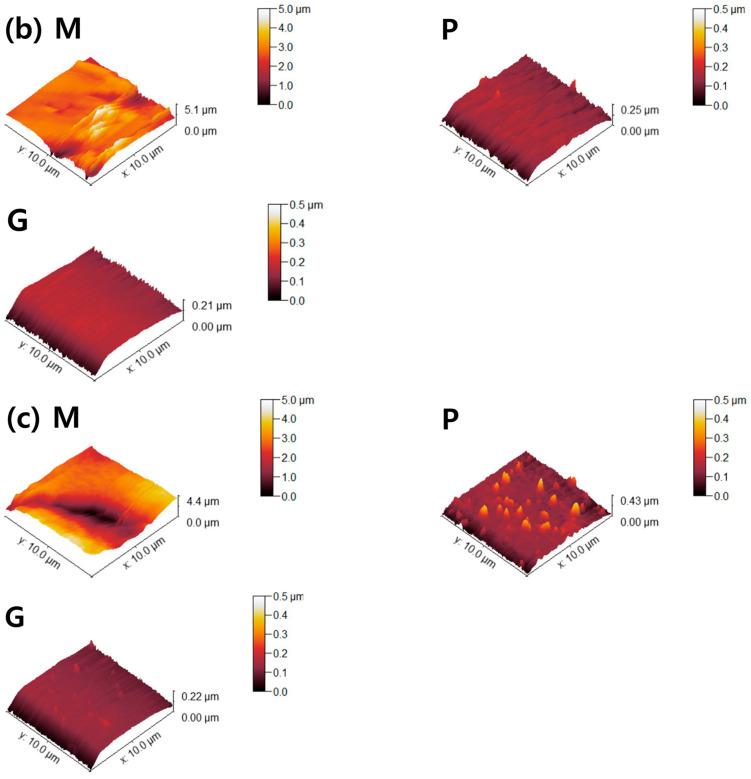
AFM topographies of (**a**) 4Y-TZP, (**b**) AM, and (**c**) AC ceramics. M, P, and G represent machining, polishing, and glazing, respectively.

The *Ra* and *Sa* of the machined 4Y-TZP decreased after polishing (Figure 5c). Surface roughness parameters were obtained from AFM topography data using the analysis software Gwyddion. In addition to the arithmetic mean roughness (*Ra* and *Sa*), the maximum height parameters (*Rz* and *Sz*) were calculated to better describe the peak-to-valley features of the surfaces. In particular, the profile parameter *Rz* was determined in accordance with ISO 21920-2 [37], and the areal parameter *Sz* was determined according to ISO 25178-2 [38]. Prior to analysis, the AFM images were plane-leveled and detrended to remove background curvature. As summarized in Table 1, all machined samples showed decreases in *Ra*, *Sa*, *Rz*, and *Sz* after polishing or glazing, regardless of the measuring method. Measurement methods using stylus profilometry with fixed cut-off filters have limitations in using various surface topographies because the size of the cut-off filter is highly dependent on the spacing of the surface topography. In 2D profilometry measurements of single surfaces, variance has been reluctantly accepted; however, *Sa* and *Sz* have been generally accepted as measures of surface quality [39,40]. The AFM surface topography is shown in Figure 6. The null hypothesis that surface treatment does not affect the *Sa* of ZrO_2_ and LD glass ceramics was rejected (*p* < 0.001). *Sa* was influenced by surface treatment ($\eta_{p}^{2}=0.699$, *p* < 0.001) and not by material type ($\eta_{p}^{2}=0.224$, *p* = 0.102). Moreover, it was not influenced by the interaction between material type and surface condition ($\eta_{p}^{2}=0.362$, *p* = 0.075).

### 3.3. In Vitro Assay

#### 3.3.1. Cytotoxicity and Cell Proliferation

The cytotoxicity of the 4Y-TZP, AM, and AC ceramics under different surface conditions was determined. For all samples, the viabilities of the L–929 and HGF-1 cells were >89% that of the negative control, as listed in Table 2. No cytotoxicity was observed in the samples under the conditions used in this study, suggesting that all the samples exhibited cytocompatibility.

**Figure 7:**
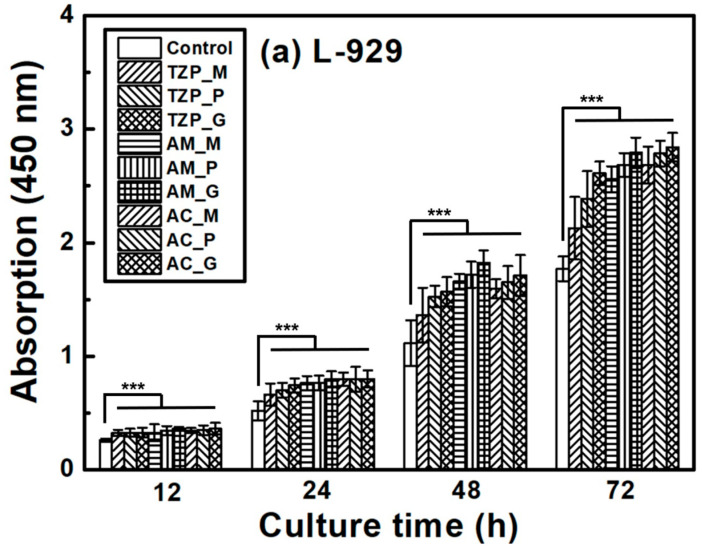

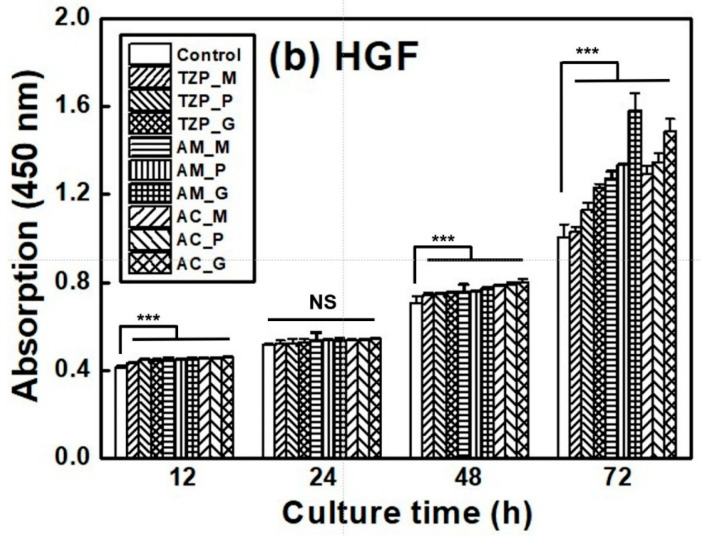
Proliferation of (**a**) L–929 and (**b**) HGF-1 cells on the negative control, 4Y–TZP, AM, and AC ceramics over time. Statistical significance is indicated by *** (*p* < 0.001). The horizontal line (NS) indicates not statistically significant (*p* > 0.05). Note: M, P, and G represent machining, polishing, and glazing, respectively.

The L–929 and HGF-1 cell proliferation results are presented in Figure 7, which shows that the cells were well attached to the ceramics and proliferated over time. The null hypothesis that surface treatments do not affect the cell proliferation of ZrO_2_ and LD glass ceramics was rejected (*p* < 0.001). Despite differences in surface condition, no significant differences in L–929 cell proliferation were observed up to 12 h after cell culture. After 24 h, the growth rate of the LD ceramics began to slightly change compared with that of ZrO_2_. The proliferation rates of the ceramics increased in the order of 4Y–TZP, AM, and AC after 72 h of culture. The proliferation of HGF-1 cells was also examined. Similarly to L–929 cells, HGF-1 cells showed no significant differences in proliferation across the different surface conditions during culture for up to 24 h. The growth rates of the glazed samples were significantly superior, and glazed LD samples grew faster than glazed 4Y-TZP samples. When glazed LD samples were cultured for 72 h, HGF-1 proliferation dramatically increased (Figure 7b).

#### 3.3.2. Live/Dead Cell Assay

**Figure 8:**
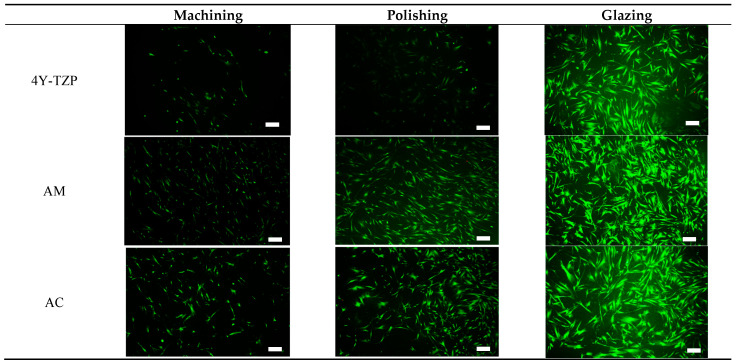
Live/dead assays of HGF-1 cells seeded onto various 4Y–TZP, AM, and AC ceramics. Note: scale bar is 300 μm.

HGF-1 cell viability and proliferation were further examined during a 72-h incubation period using a live/dead assay kit [26,27,28,30,31]. Viable (green staining) and dead (red staining) HGF-1 cells were visualized using a fluorescence microscope. As shown in Figure 8, viable green-stained HGF-1 cells were primarily observed throughout the samples, with significant cell attachment and growth observed in the glazed samples. The lowest cell density was observed in machined and polished 4Y–TZP owing to its higher hydrophobicity and stiffness. Machined LD ceramics had the lowest cell density; however, for polished LD ceramics, the WCA decreased, indicating a slight increase in cell density. As shown in Figure 5a, as the hydrophilicity increased, cell adhesion, proliferation, and spreading were enhanced in the glazed samples, regardless of material type.

### 3.4. Biofilm Thickness and Biomass Measurements

**Figure 9:**
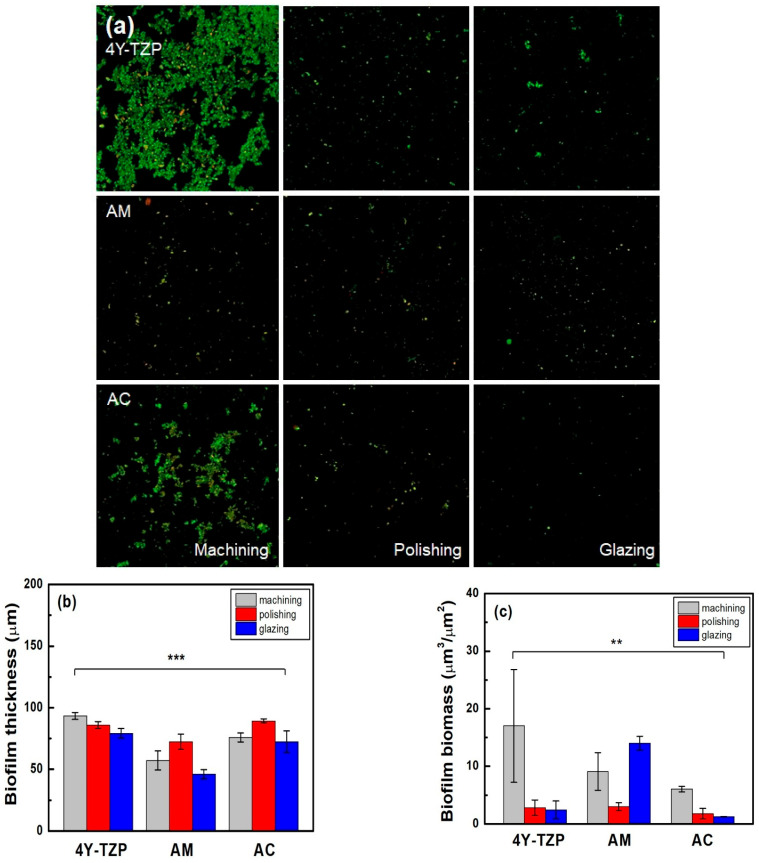
(**a**) Live/dead staining images of bacteria attached to the surfaces of the specimens. Quantitative analysis of the (**b**) thickness and (**c**) biomass of the biofilms. Statistical significance is indicated by ** (*p* < 0.01) and *** (*p* < 0.001).

The number of viable bacteria attached to 4Y-TZP, AM, and AC stained with green fluorescent dye was higher in the machined group than in the other groups (Table 2 and Figure 9a). As shown in Figure 9b, the lowest biofilm thickness was observed in all glazed samples. Subsequently, quantitative analyses were performed using biofilm biomass. The null hypothesis that surface treatments do not affect the biofilm biomass of ZrO_2_ and LD glass ceramics was rejected (*p* < 0.01). For glazed AM, higher biomass values of the biofilms were observed. Thus, because all specimens were polished, the biomass of the biofilm decreased (Figure 9c). Overall, the glazed 4Y–TZP and AC samples showed a significant bacterial inhibition effect compared to the other groups [20,21,22,23,32].

## 4. Discussion

The success of dental implants depends on the stable attachment and efficient sealing between the implant surface and surrounding soft tissue and alveolar bone [12,13]. As shown in Figure 1, the surface of the ZrO_2_ coping and LD crown of AC can affect the barrier between epithelial cells and fibroblasts at the interface of the dental crown and epithelial soft tissue. Soft tissue cells, such as the junctional epithelium (HGF-1) and connective tissue (L–929), may compete with bacteria during dental restoration. Improving the soft tissue adhesion may reduce bacterial adhesion, thereby reducing the risk of periodontitis [7]. Attaching a firm epithelium and connective tissue to the mucosal part of the implant can prevent apical migration of the epithelial tissue [34,35]. When the soft tissue is firmly attached to the mucosal part of the implant or abutment, it functions as a biological barrier, preventing the invasion of bacterial toxins and providing resistance to alveolar bone resorption [13,34,41,42,43,44,45]. Among the tested samples, 4Y–TZP exhibited a lower cell proliferation rate compared to LD, suggesting that LD is more favorable in generating soft tissue responses. Furthermore, Figure 7 shows that the LD glaze treatment may be more effective than ZrO_2_ in promoting the proliferation of oral fibroblasts and epithelial cells (*p* < 0.0001) [12,25]. Thus, surface hydrophilicity (Table 1 and Figure 4) may significantly influence cell proliferation [7,46,47,48].

Two parameters associated with cell adhesion to surfaces in contact with gingival tissue are surface roughness and wettability [5]. In this study, LD glass ceramics were prepared in a crystallized form through machining, polishing, and glazing, and 4Y–TZP was used as a reference. 4Y–TZP has no potential toxic effects and results in healthy soft tissue surrounding the implants [1,7,8,9,10,49,50,51,52]. Polished 4Y–TZP samples with a mirror finish did not provide any advantage over samples within the roughness threshold (approximately *Ra* = 0.2 μm) that balances bacterial adhesion and soft tissue attachment in clinical studies [7,11,12,17]. Rimondini *et al.* [11] reported that no significant differences in bacterial colonization were observed between ZrO_2_ samples with an *Ra* of <0.2 μm owing to soft tissue sealing. According to the AFM results (Figure 6), the *Sa* of the machined 4Y-TZP was 125.79 ± 69.29 nm, while those of the machined AM and AC were 246.72 ± 69.29 and 395.25 ± 69.29 nm, respectively. However, polishing or glazing significantly improved the *Sa* and *Sz*. The *Sa* and *Sz* of the machined AC decreased after polishing and glazing. Thus, surface treatment significantly affected the *Sa* and *Sz* of the samples. Consequently, surface treatment has a significant effect on the *Sa* and *Sz* of dental ceramics [39,40].

After glazing, the WCA of the polished 4Y–TZP surface decreased from 71.3° to 21.9°, indicating an increase in hydrophilicity [5]. As hydrophilicity increases, free water exists near the surface, which may hinder protein adsorption [17,21,22,23]. Notably, cell proliferation and bacterial attachment are opposing phenomena [14,16]. In this study, the WCA of polished 4Y–TZP was the highest, whereas glazed AC showed the lowest WCA (14.0°). The experimental *Sa* results of the glazed samples were similar. For example, the WCA sharply decreased after glaze treatment. The decrease in the WCA of the LD sample was attributed to its less rigid structure comprising crystalline and glassy phases compared to that of fully crystallized 4Y–TZP (Figure 5) [2]. Thus, glazing was more beneficial for wettability. The rejection of the null hypothesis that material type and surface conditions have no significant effect on the WCA of dental ceramics suggests that glazing has a major impact on wettability.

The mechanical properties of restorations and dental materials are also affected by the moisture present in the oral cavity [25]. The oral cavity is extremely heterogeneous and contains a diverse microbial community of hundreds of species of bacteria, which colonize the hard surfaces of teeth and soft surfaces such as the tongue and buccal mucosa [36,53,54,55]. To overcome adverse effects such as gingivitis and periodontitis, efforts have been made to reduce bacterial adhesion through surface modifications, such as the machining, polishing, and glazing of ceramics [5,10,11,12,13,15,16,53,54,55]. The mechanical properties can be degraded by surface scratches introduced during machining, moisture ingress resulting from microcracks, or pore exposure during firing [25]. Glazing aims to improve the color matching and natural appearance of restorations by simulating enamel. The glazed specimens form a robust coating on the surface that resists scratches that occur during polishing, fills the micropores, and prevents moisture penetration [25]. Glazes typically comprise silica, porcelain, glass ceramics, or heavy crystalline solids. The glazing technique provides a smooth surface with strong hydrophilicity owing to the abundance of hydrophilic silica [25,56,57]. This treatment is highly effective for reducing the adhesion of biomaterials and preventing biofilm formation on dental surfaces in oral bacterial environments.

Excellent wettability and HGF-1 responses (Table 1 and Table 2 and Figure 5 and Figure 8) were observed in the glazed ceramics, regardless of the material type. Previously, we demonstrated that 4Y–TZP and LD ceramics were not cytotoxic in vitro. Glaze treatment is favorable for the adhesion, spread, and proliferation of HGF-1 cells, rendering it suitable for application in dental prosthetics. In addition, glazed specimens exhibit a coating layer that fills scratches, cracks, and defects that may occur during processing [19,25,48,49]. High stresses that occur during processing can result in the formation of surface cracks, which can reduce the strength and reliability of the material [7,19,50,51]. Glazing has the advantages of inhibiting moisture penetration, improving mechanical properties, and eliminating surface pores. The durability of glazed dental prostheses can be improved through cell adhesion and proliferation, and strength degradation can be delayed by reduced exposure to oral moisture [25]. Among the surface treated specimens, the lowest WCA and bulk density were observed in the glazed specimens, suggesting that wettability is an important factor for cell adhesion and proliferation. Therefore, the application of glazes to dental ceramics can improve cell adhesion and proliferation.

Biofilm-related infections tend to progress to chronic diseases due to the resilience of oral bacteria embedded within an extracellular polymeric substance matrix that protects them from antibiotics and host defenses [14]. In the oral cavity, biofilm that is formed on restorative materials can result in secondary caries, periodontitis, and ultimately tooth loss if left untreated. Furthermore, persistent biofilms can serve as reservoirs for pathogenic microorganisms, which can enter the bloodstream and cause systemic infections or embolic complications [14,41,42,43,44]. Enzymes such as glycoside hydrolases, deoxyribonucleases, and proteases can degrade the biofilm matrix and release sessile bacteria into planktonic form, thereby enhancing their susceptibility to antibiotics and the host immune response [14,41,42,43,44].

Considering the clinical relevance of the oral environment, we evaluated the thickness and biomass of biofilms formed by human saliva on samples with different surface treatments. The glazed 4Y–TZP and AC samples exhibited biomass values of 2.4 ± 1.5 and 1.2 ± 0.0 μm^3^/μm^2^, respectively, which were lower than that for AM (14.0 ± 1.1 μm^3^/μm^2^). Consistent with previous studies, glazing treatment resulted in lower surface energy, which contributed to reduced bacterial adhesion. The observed low biomass values indicate strong resistance to biofilm formation, likely due to hydration shell formation and steric repulsion effects that may delay bacterial attachment [20,21,22,23,41,42,43,44,58]. However, glazing may also increase surface porosity or introduce localized surface defects, potentially facilitating bacterial attachment in certain materials (Table 1 and Table 2). This dual effect underscores the importance of optimizing glazing protocols to minimize undesirable surface characteristics. The rejection of the null hypothesis that the surface conditions do not significantly affect biofilm formation on dental ceramics suggests that surface treatments have a beneficial effect on bacterial resistance. A key limitation of this study is that it was conducted under in vitro and ex vivo conditions. While in vitro and ex vivo models offer valuable initial insights, they cannot perfectly replicate the dynamic interactions of the oral environment, including salivary secretion, immune responses, and dietary variations. Therefore, future in vivo and clinical studies are warranted to validate the long–term antibacterial performance and clinical relevance of glazed surfaces in restorative materials.

## 5. Conclusions

The surface modification, such as machining, polishing, and glazing, of dental ceramics effectively influences their biological performance. Glazing significantly reduced the WCA and bulk density of dental ceramics, thereby enhancing their porosity and surface wettability. Consequently, glazed surfaces promoted HGF-1 and L–929 fibroblast attachment and proliferation, suggesting improved soft-tissue sealing around dental implants. Moreover, the glazed 4Y–TZP and AC samples exhibited lower biofilm biomass, indicating enhanced bacterial resistance compared to machined or polished surfaces. Although polishing resulted in an improved cellular response and reduced biofilm formation compared to machining, glazing demonstrated superior efficacy. Thus, optimized glazing treatment on the surface of dental ceramics can potentially prevent peri-implant diseases, improving implant restoration prognosis. Despite these promising in vitro and ex vivo results, further in vivo and clinical studies are necessary to confirm the long-term biological and antibacterial effectiveness of glazing. 

## Figures and Tables

**Table 1:** Physical properties of the various samples after surface treatment.

Specimens	Surface Condition	*Ra* (μm) (Profilometer)	*Sa* (μm) (AFM)	*Rz* (μm)	*Sz* (μm)	WCA (°)	Bulk Density (g/cm^3^)	Apparent Porosity (%)
4Y-TZP	Machining	0.26 ± 0.047	0.12 ±0.069	1.75 ± 1.627	1.50 ± 1.186	68.9 ± 5.2	6.04 ± 0.01	0.42 ± 0.01
	Polishing	0.01 ± 0.001	0.02 ± 0.008	0.31 ± 0.183	0.31 ± 0.186	71.3 ± 8.9	6.04 ± 0.02	0.60 ± 0.11
	Glazing	0.05 ± 0.001	0.03 ± 0.007	0.55 ± 0.315	0.54 ± 0.303	21.9 ± 1.9	5.42 ± 0.03	2.22 ± 1.19
AM	Machining	0.79 ± 0.054	0.24 ± 0.056	5.12 ± 1.650	3.42 ± 0.916	62.9 ± 3.4	2.39 ± 0.01	2.87 ± 0.98
	Polishing	0.01 ± 0.006	0.03 ± 0.011	0.49 ± 0.220	0.49 ± 0.217	56.7 ± 4.7	2.40 ± 0.01	1.26 ± 0.82
	Glazing	0.05 ± 0.001	0.02 ± 0.002	0.37 ± 0.216	0.23 ± 0.039	25.4 ± 3.9	2.38 ± 0.03	3.58 ± 1.72
AC	Machining	0.80 ± 0.054	0.39 ± 0.237	2.87 ± 1.357	2.80 ± 1.330	58.4 ± 4.0	2.42 ± 0.02	1.78 ± 0.78
	Polishing	0.01 ± 0.001	0.04 ± 0.014	0.75 ± 0.502	0.61 ± 0.276	46.8 ± 13.6	2.43 ± 0.02	1.68 ± 0.66
	Glazing	0.05 ± 0.001	0.02 ± 0.004	0.29 ± 0.089	0.29 ± 0.066	14.0 ± 3.2	2.38 ± 0.01	2.29 ± 0.35

**Table 2:** Biological properties of the various samples after surface treatment.

Specimens	Surface Condition	HGF-1 cell Viability (%)	L-929 Cell Viability (%)	Biofilm Biothickness (μm)	Biofilm Biomass (μm^3^/μm^2^)
4Y-TZP	Machining	96.9 ± 2.4	91.3 ± 1.1	93.3 ± 2.8	17.0 ± 9.7
	Polishing	99.4 ± 3.1	91.7 ± 2.4	85.8 ± 2.8	2.7 ± 1.3
	Glazing	99.5 ± 4.5	90.9 ± 1.6	79.1 ± 3.8	2.4 ± 1.5
AM	Machining	92.5 ± 0.4	92.8 ± 2.6	57.1 ± 7.8	9.0 ± 3.2
	Polishing	90.9 ± 1.0	92.1 ± 6.5	72.3 ± 6.1	2.9 ± 0.6
	Glazing	89.0 ± 0.5	91.9 ± 4.1	46.0 ± 3.6	14.0 ± 1.1
AC	Machining	100.4 ± 3.5	97.0 ± 2.1	75.8 ± 3.6	6.0 ± 0.4
	Polishing	100.5 ± 1.2	96.5 ± 2.2	89.2 ± 1.7	1.7 ± 0.9
	Glazing	98.7 ± 0.9	96.3 ± 4.0	72.3 ± 8.8	1.2 ± 0.0

## Data Availability

The original contributions presented in this study are included in the article material. Further inquiries can be directed to the corresponding author.

## Abbreviations

The following abbreviations are used in this manuscript:

LD	lithium disilicate
AM	Amber^®^ Mill
AC	Amber^®^ Mill Abut-Crown
HGF-1	human gingival fibroblast
WCA	water contact angle
*Ra*	arithmetic average roughness
*Sa*	arithmetic mean surface height
*Rz*	average maximum height of the profile
*Sz*	maximum height
$\eta_{p}^{2}$	partial eta-squared
4Y-TZP	4 mol% Y_2_O_3_-stabilized tetragonal ZrO_2_ polycrystal
DW	distilled water
NS	not statistically significant
PTFE	Polytetrafluoroethylene
PBS	phosphate-buffered saline
ANOVA	analysis of variance
SEM	scanning electron microscopy
AFM	atomic force microscopy
